# The additional value of home exercises to self-management for the treatment of masticatory muscle pain. A clinical trial

**DOI:** 10.4317/jced.61549

**Published:** 2024-06-01

**Authors:** Diego De Nordenflycht, Catalina Díaz, Javier Salinas, Héctor Toloza

**Affiliations:** 1 DDS, MSc. Associate Professor, Faculty of Dentistry, Universidad Andres Bello, Viña del Mar, Chile; 2 DDS. Private practice, La Calera, Chile; 3 DDS. Instructor, Faculty of Dentistry, Universidad Andres Bello, Viña del Mar, Chile

## Abstract

**Background:**

Initial management of temporomandibular disorders (TMD) based on self-management (SM) is strongly recommended by literature, nevertheless, research is needed to investigate the efficacy of different types of interventions under each component of SM against each other for the management of particular subtypes of TMD. The present study aimed to compare the clinical effectiveness of SM and SM with additional mandibular home exercises for the management of myalgia of masticatory muscles.

**Material and Methods:**

A clinical trial was conducted with 54 subjects with a diagnosis of myalgia according to the Diagnostic Criteria for Temporomandibular Disorders (DC/TMD), which were randomised into two groups: treated with SM (SM group) and treated with SM and mandibular home exercises (SM+EX group). Follow-ups were carried out at 2, 6, and 10 weeks, where it was evaluated: pain in the masticatory muscles, jaw opening range of motion, and mandibular functional limitation. Data were analysed using Wilcoxon signed-rank test for comparisons between periods (baseline, and weeks 2, 6, and 10) and Wilcoxon rank-sum test for comparison between groups (*p*=0.05).

**Results:**

All the variables showed significant improvement (*p*<0.05) from baseline to the first follow-up and were maintained later, i.e. both groups were able to reduce pain, increase the jaw opening range of motion, and improve the mandibular functional limitation, although no significant differences were found between groups (*p*>0.05).

**Conclusions:**

The self-management program was able to reduce pain intensity, increase the jaw opening range of motion and improve functional limitation, but the addition of mandibular home exercises do not have a significant impact on myalgia of the masticatory muscles in the short-term.

** Key words:**Myalgia, Self-care, Self-management, Temporomandibular joint disorders.

## Introduction

Temporomandibular disorders (TMD) are a group of commonly occurring oro-facial pain conditions, which affect the temporomandibular joint, the masticatory muscles or both (1). According to the current Diagnostic Criteria for Temporomandibular disorders (DC/TMD) taxonomy (2), myalgia refers to pain of muscle origin that is affected by jaw movement, function, or parafunction, and replication of this pain occurs with provocation testing of the masticatory muscles. Myalgia is the most common TMD diagnosis and occurs in about 80% of patients with TMD (3).

Patients with myalgia of masticatory muscles seek treatment to a greater extent than patients with TMJ arthralgia (4), frequently have a negative impact over quality of life (5), and, therefore, this chronic pain requires specific care and treatment (6). Several treatments have been proposed to manage myalgia of masticatory muscles including: self-management, cognitive-behavioural therapy, physiotherapy, postural therapy, jaw exercises, manual or physical treatment such as acupuncture, dry needling, and wet needling therapies, transcutaneous electrical nerve stimulation, thermal therapy, occlusal appliances, and drug therapy, among others (7). Nevertheless, the majority of cases respond well to simple reversible therapies (1).

Self-management (SM) or self-care comprises a core of non-invasive initial therapy that may include education on TMD (and analgesia usage), self-monitoring advice for habits, relaxation and posture training, relaxation strategies, and jaw exercises (8). Initial management of TMD based on SM is strongly recommended in systematic reviews (1,9,10). Nevertheless, it has been suggested that more studies are needed to investigate the efficacy of different types of interventions under each component of SM against each other for management of particular subtypes of TMD (8); also, there is a need to explore early management of chronic orofacial pain in primary care using SM interventions, using standardised outcome measures so that they can be comparable across trials (9). Based on the above information, the aim of the present study is to compare the clinical effectiveness of SM and SM with additional mandibular home exercises for the management of myalgia of the masticatory muscles. The hypothesis proposed is that adding home-exercises to SM generates a greater improvement on pain in subjects with myalgia.

## Material and Methods

-Study design. A randomised clinical trial was conducted at the general practice Dental Clinic of Andrés Bello University (Viña del Mar, Chile). The study subjects were recruited from the patients seeking treatment for jaw pain at the university dental clinic. All subjects were informed about the study by their operator and gave their written consent before initiating the study.

-Sample Size Calculation. The sample size was calculated using G*Power software considering an effect size d = 0.8, an α-error = 0.05, and a power (1-β) = 0.80. An expected loss of 10% was established. Finally, the minimum number of participants estimated for each group was 23 subjects (46 in total).

-Inclusion and Exclusion Criteria. Female and male adults who needed treatment for TMD-related myalgia of the masticatory muscles, diagnosed using the DC/TMD protocol were included. A total of 70 subjects were examined, of which 54 men and women, without distinction of gender, were eligible and included in the study; none rejected their participation. The subjects’ inclusion criteria were: aged between 18 and 40 years, with presence of masticatory muscle myalgia according to DC/TMD criteria. The exclusion criteria were: painful arthrogenous TMD (e.g. arthralgia, osteoarthritis, arthritis), locking jaw, history of TMD treatment, recent history of facial or cervical trauma, ongoing orthodontic treatment, abnormal dental mobility, subjects with loss of more than two teeth other than third molars and/or premolars due to orthodontic indication; subjects with systemic musculoskeletal diseases or comorbid pain sites elsewhere in the body; subjects who are under analgesic treatment; subjects with a diagnosed intellectual disability who cannot express their will to participate in scientific research as established by law No. 20,584 of Chile.

-Randomization and interventions. After meeting the inclusion and exclusion criteria, the subjects were assigned to one of two groups following a randomization procedure through a computationally generated sequence, “list randomizer” by random.com. The established groups were:

1. Self-management group (SM group). This group consisted of 27 subjects who received a scheme of SM program consisting of verbal and written information on the aetiology and prognosis of TMD. In addition, advice on habits and behaviour changes, relaxation techniques, sleep hygiene, diet modification, thermotherapy, encouragement to practise social and aerobic activities, and how to prevent risk factors and bad habits. For this purpose, the patient was sitting on a dental chair in a comforTable position with a laptop to display audio-visual material that supported the verbal information. Attention was paid to ensure that the subject is fully aware of the given information.

2. Self-management plus home exercises group (SM+EX group). This group consisted of 27 subjects who received the SM program described above in combination with a mandibular home exercise routine. Home exercises therapy includes self-massage of the masseter (by slight rolling movements performed with the index, middle and ring fingers located extra-orally over the muscle area and the thumb placed intra-orally exerting counter pressure) and temporalis muscles (by slight circular movements performed with the ipsilateral index, middle and ring fingers on the muscle area), self stretching (by slowly open the mouth until an initial pain sensation is experienced, then, mouth opening is gently forced using the thumb in upper teeth and index finger in lower teeth), and masticatory muscles strengthening exercise (by performing active mouth opening movements against hand resistant).

-Sequence of interventions. Treatment of the subjects consisted of 5 sessions. The patients were contacted by telephone the day before every session appointment to avoid drop-outs and lags in monitoring the clinical symptoms. Also, subjects from the SM+EX group were asked to keep their exercise “patient diary”. The sessions` protocol was the following: (S1) diagnosis of recruited subjects and personal data such as sex, age, and ID number were registered; (S2) three days after S1, SM instruction and explanation of the follow-up sessions were delivered to all subjects, and additionally home-based exercises were instructed and explained for the SM+EX group only; (S3) first control and SM (or SM and exercises) reinforcement at 2 weeks; (S4) second control and SM (or SM and exercises) reinforcement at 6 weeks; (S5) third control and it was encouraged to continue with SM, and assist to periodical controls with their dentist. Over-the-counter 500mg paracetamol was allowed during the period of the trial. All subjects were told that in case of complications, they should contact the operator. Referral to a TMD specialist was made if initial symptom remission was not achieved after the trial period, or if worsening of the initial symptoms was reported.

**Figure 1:**
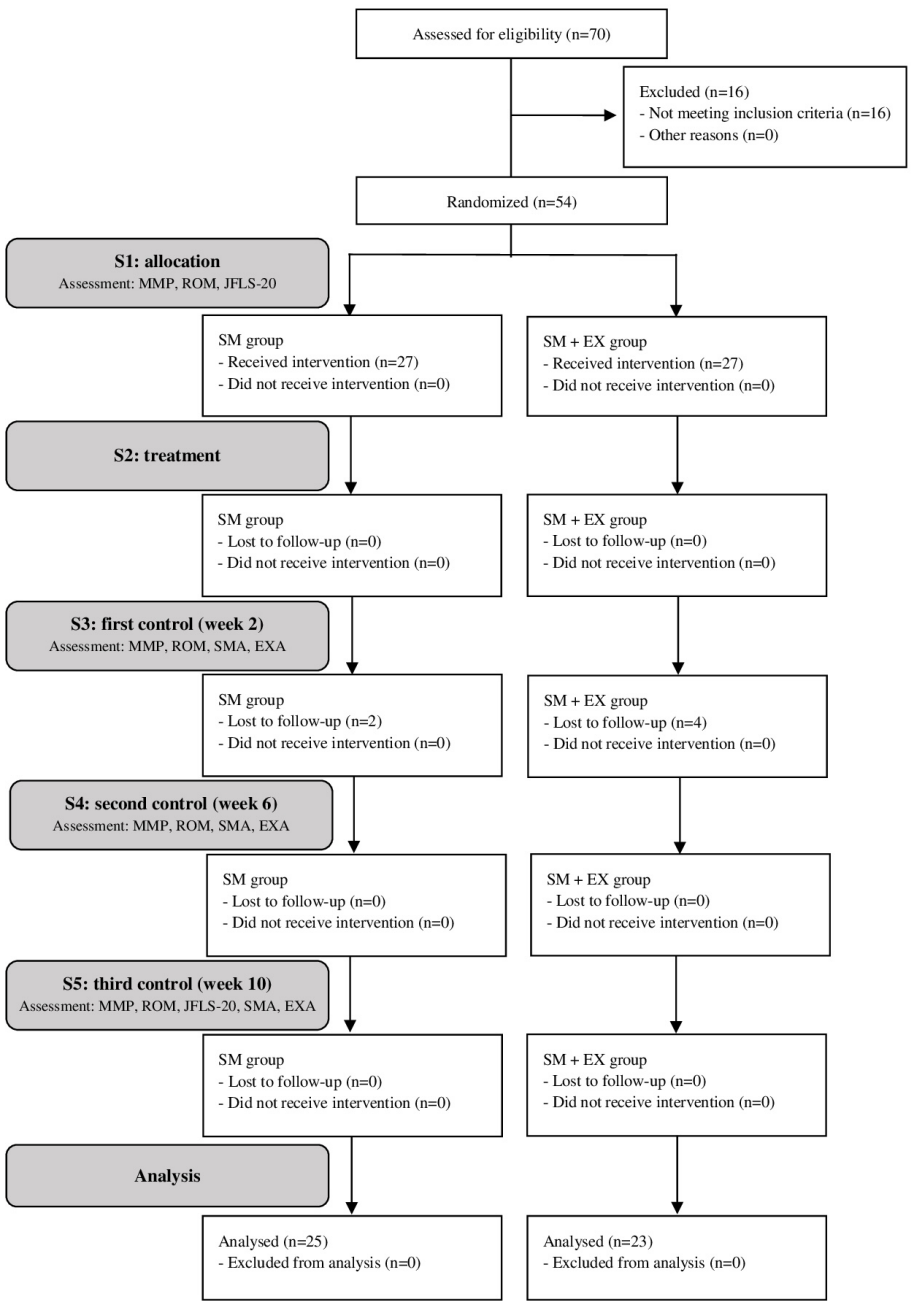
Flow chart of the selection of subjects referred for TMD treatment. SM: self-management; EX: home exercises; MMP: masticatory muscle pain; ROM: jaw opening range of motion; JFLS-20: Jaw functional limitation scale; SMA: self-management adherence; EXA: home exercises adherence.

-Assessment methods. The initial evaluation was carried out following the symptom questionnaire and clinical examination guidelines according to the DC/TMD protocol (2) and the Graded Chronic Pain Scale (GCPS) of axis II of the DC/TMD were applied at baseline. An intraoral clinical examination was also performed to exclude dental causes of pain. The assessments were done with the examiner blinded to group allocation at all time points. The Subject’s flow chart is shown in Figure 1. The following variables were analysed:

1. Masticatory muscle pain (MMP). Pain intensity was measured using a verbal numeric scale; subjects were asked to rate the pain intensity using a numerical rating scale where “zero” corresponded to “no pain”, and “ten” corresponded to “extremely strong pain”. MMP was assessed in the initial evaluation, and weeks 2, 6, and 10.

2. Jaw opening range of motion (ROM). Autonomous and comfortable maximum mouth opening, without feeling pain and non-assisted by the operator, measured in millimetres from upper incisal edge to lower incisal edge, compensating the overbite. ROM was assessed in the initial evaluation, and weeks 2, 6, and 10.

3. Jaw functional limitation (JFLS-20). The assessment of the global functional limitation in terms of mastication, mobility, and communication of subjects was achieved with the survey of jaw functional limitation scale (JFLS-20) of axis II of the DC/TMD (2). The global score was obtained calculating the mean value of mastication, mobility, and communication items. JFLS-20 was assessed in the initial evaluation and week 10.

4. Adherence to self-management (SMA). Qualitative assessment of the adherence (or engagement) of subjects reported to the SM program that was categorised as “good” (⅔ of the time), “moderate” (between ⅔ and ⅓ of the time), and “deficient” (less than ⅓ of the time). SMA was assessed at weeks 2, 6, and 10.

5. Adherence to home exercises (EXA). Qualitative assessment of the adherence (or engagement) of subjects according to the “patient diary” of home exercises that was categorised as “good” (⅔ of the time), “moderate” (between ⅔ and ⅓ of the time), and “deficient” (less than ⅓ of the time). EXA was assessed only in the SM+EX group at weeks 2, 6, and 10.

-Statistical analysis. Data were analysed using Wilcoxon signed-rank test for comparisons between periods and Wilcoxon rank-sum test for comparisons between groups of the variables MMP, ROM, and JFLS-20. SMA was analysed using Fisher exact test. EXA was descriptively reported. The level of significance was established at 0.05. All statistical analysis was performed using the R-Cran 3.01 software.

## Results

The data that support the findings of the study and Consort checklist are openly available in Open Science Framework (https://osf.io/efbc2). The trial initiated with 54 subjects that met the inclusion criteria, and 48 of them completed the 10 weeks treatment. Regarding subjects´ gender, more females (n=35) than males (n=13) were recruited whose distribution was homogeneous between groups (Fisher Exact Test *p*=0.749). Age of subjects was slightly superior in the SM group (27.6 ± 5.9 years) than the SM+EX group (24.3 ± 4.2 years), but this difference was not statistically significant (Wilcoxon rank-sum test *p*=0.101). Regarding GCPS, a greater number of subjects with grade II (high intensity pain without disability; n=27), and grade I (low intensity pain without disability; n=15) was found, although the difference between groups was not statistically significant (Fisher Exact Test *p*=0.312).

-Masticatory muscle pain (MMP). Mean values of MMP are shown in Table 1. In the SM group, a reduction of MMP was observed between baseline and week 2 (28%), 6 (57%) and 10 (85.7%), whose differences were statistically significant (*p*=0.001, *p*=0.000, and *p*=0.000 respectively). In the SM+EX group, a reduction of MMP was observed between baseline and week 2 (27%), week 6 (47%) and week 10 (78%), whose differences were statistically significant (*p*=0.001, *p*=0.000, and *p*=0.000 respectively). No statistically significant differences were found between groups (*p* >0.05).

-Jaw opening range of motion (ROM). Mean values of ROM are shown in  Table 1. In SM group, an increase of ROM was observed between baseline and week 2 (9%), 6 (22%) and 10 (31.4%), whose differences were statistically significant (*p*=0.002, *p*=0.000, and *p*=0.000 respectively). In SM+EX group, an increase of ROM was observed between baseline and week 2 (12%), week 6 (23%) and week 10 (34%), whose differences were statistically significant (*p*=0.014, *p*=0.001, and *p*=0.000 respectively). No statistically significant differences were found between groups (*p* >0.05).

-Jaw functional limitation (JFLS-20). Mean values of JFLS-20 are shown in  Table 1. In the SM group, a reduction of JFLS-20 score was observed between baseline and week 10 (66.8%), which difference was statistically significant (*p*=0.000). In the SM+EX group, a reduction of JFLS-20 score was observed between baseline and week 10 (75.9%), which difference was statistically significant (*p*=0.000). No statistically significant differences were found between groups at week 2 (*p*=0.657) and week 10 (*p*=0.671).

-Adherence to self-management (SMA). Mean values of SMA are shown in  Table 1. A higher percentage of “good” adherence to SM was observed at week 10 than week 2 and 6. A greater adherence to SM was observed for the SM group than the SM+EX group in each period, but these differences were not statistically significant (*p*>0.05).

-Adherence to home exercises (EXA). Mean values of EXA are shown in  Table 1. Adherence to home exercises decreased over time, but most subjects of the SM+EX group achieved a good adherence in each period. No adverse effects were reported after treatment for both groups.

## Discussion

The present trial compared the clinical effectiveness of SM and the addition of home exercises in the treatment of myalgia of the masticatory muscles over a 10 weeks period. All assessed variables showed significant improvement from baseline to the first follow-up and were maintained later, i.e. both groups reduced pain intensity, increased the mandibular range of motion, and improved the mandibular functional limitation, although no significant differences were found between SM and SM plus home exercises groups.

Similar results have been found for SM versus home exercises for myogenous TMD (11-13), although, in general practice, therapeutic exercises are considered effective in the management of muscular TMD (9,14-16). Most available evidence that supports SM and home exercise for myogenous TMD uses RDC/TMD or other less prevalent diagnostic taxonomies (1,9,14-16), so the results of the present study, which includes subjects with the diagnosis of “myalgia” according to DC/TMD, might not be completely comparable to the aforementioned trials. For the diagnosis of “myalgia”, the DC/TMD does not differentiate between primary and secondary origin such as ICOP (17), or its chronicity. Given the above, differentiation between acute and chronic TMDs may influence its management: while patients with acute TMDs often require simple explanation, SM and analgesics, patients with chronic TMD require complex chronic pain medications and ongoing chronic pain management support (18).

The addition of home exercises did not achieve better results than the SM program alone in terms of jaw opening range of motion and mandibular functional limitation, which is consistent with the results from a recent systematic review that found that home exercises have a mild to moderate effect on TMD compared to other conservative treatments (19). Moreover, considering that initial assessment of GCPS show that most subjects have “no disability” it could be expected that exercises that aimed to improve mandibular mobility would not have a significant impact on the subjects.

SM forms the basis of acute and chronic TMDs management. It is best described as a patient-focused approach that aims to facilitate a patient’s understanding of their condition and ability to work successfully with health care professionals to help manage their condition (18). SM efficacy for TMD treatment has been previously assessed (1), but most of these trials used the SM as a control group or some form of basic standardised treatment to which other forms of interventions are adhered in order to test its efficacy (1,20). It has been suggested that structured self-management may be more beneficial than usual treatment (involving physiotherapy, education, medication, or intraoral flat-plane occlusal appliances) for the management of persistent TMD (21) which is consistent with present results.

A recent Delphi study identified the main components that constitute the core of a SM programme for TMD: education; jaw exercises; massage; thermal therapy; dietary advice and nutrition; and parafunctional behaviour identification, monitoring and avoidance (8). Notwithstanding, it might be possible that incorporating numerous behaviour change techniques would make a SM programme too intensive, providing the patient with too much information or too many tasks which would then potentially decrease adherence to the programme (1), therefore the need arises to investigate different types and/or techniques of each individual component of SM against each other for management of particular subtypes of TMD.

The adherence (or engagement) to SM varied in time and between groups, although these differences were not significant. It was observed that SM adherence fluctuated during the trial as it was higher at week 10 than week 2; also, adherence to home exercises fluctuated during the trial, and it was higher at week 2 than week 10. It has been advised that SM programmes should be reviewed to ensure comprehension and adherence since they rely on a therapeutic alliance between clinician and patient and therefore require patient comprehension, motivation, cooperation, active participation, and adherence (8). The processes of engagement with a SM program is supported in key mechanisms of change centred around: identification with the intervention, feeling believed and understood, obtaining a plausible explanation for symptoms, degree of perceived effort required to engage, acceptance of having a long-term condition, and receiving demonstrative and positive feedback (22).

There are some limitations to this study that should be considered: the absence of a negative control group (without intervention) does not allow to determine if the improvement on variables assessed were the result of the treatments or, in some degree, product of “regression to the mean” that characterises TMDs natural course (23); although DC/TMD symptom questionnaire (question two) as well as GCPS (first question) regards for how long the patient has had pain, the present study do not classify patients in acute/chronic pain, which further research should consider since acute forms of TMDs typically represent simpler cases that can be successfully managed with SM, but chronic TMDs might require more complex strategies (24); some initiatives such as the Initiative on Methods, Measurement, and Pain Assessment in Clinical Trials (IMMPACT) and the DC/TMD have recommended the assessment of not only physical features but also psychological and emotional functioning associated with chronic pain (25), which might be consider for future research; and the study design did not pay attention to whether the participants in the SM+EX group performed their home exercise or not, which may have impacted the results.

## Conclusions

The structured self-management program was able to reduce pain intensity, increase the jaw opening range of motion and improve functional limitation, but the addition of mandibular home exercises do not have a significant impact on myalgia of the masticatory muscles in the short-term. Considering the limitations of the present study, home exercises should not be discarded as a form of treatment for myogenous TMDs, although authors suggest that initial management of patients with myalgia of the masticatory muscles should be based on a simpler but structured form of self-management program.

## Figures and Tables

**Table 1:** Mean (SD) values for masticatory muscle pain, Jaw opening range of motion, mandibular functional limitation, adherence to self-management and adherence to home exercises, per time and groups.

			SM group	SM+EX group	p-values(between groups per week)
MMP (in VNS)	Baseline	-	5.16 (1.31)¶	5.22 (1.70)*	1
	Week 2	-	3.68 (2.08)¶	3.65 (1.83)*	0.8093747
	Week 6	-	2.24 (1,59)¶	2.65 (1.43)*	0.4150475
	Week 10	-	0.76 (0.78)¶	1.04 (0.93)*	0.2758765
ROM (in mm)	Baseline	-	36.76 (8.62)	35.57 (8.62)	0.5075057
	Week 2	-	39.44 (7.14)	38.74 (8.25)	0.597663
	Week 6	-	43.52 (6.55)	42.13 (7.30)	0.3139178
	Week 10	-	46.24 (5.29)	45.74 (7.50)	0.7310959
JFLS-20	Baseline	-	2.23 (1.38)§	2.49 (1.55)#	0.6572
	Week 10	-	0.70 (0.73)§	0.69 (0.59)#	0.6711
	Variation	-	66.8%	75.9%	-
SMA	Week 2	Good	56%	39%	0.07783
		Moderate	40%	57%	
		Deficient	4%	4%	
	Week 6	Good	56%	35%	0.1936
		Moderate	44%	61%	
		Deficient	0%	4%	
	Week 10	Good	72%	43%	0.682
		Moderate	28%	57%	
		Deficient	0%	0%	
EXA	Week 2	Good	-	83%	-
		Moderate	-	17%	-
		Deficient	-	0%	-
	Week 6	Good	-	57%	-
		Moderate	-	39%	-
		Deficient	-	4%	-
	Week 10	Good	-	65%	-
		Moderate	-	30%	-
		Deficient	-	4%	-

MMP: Masticatory muscle pain. ROM: jaw opening range of motion (in mm). JFLS-20: jaw functional limitation scale (0-10); SM: self-management. EX: home exercises. EXA: home exercises. SMA: self-management adherence. Same symbols (¶, *, §, #) indicates statistically significant differences (p<0.05) within groups along time for each variable.

## Data Availability

The datasets used and/or analyzed during the current study are available from the corresponding author.
